# The current utility and future use of the medical student performance record: A survey of perceptions across Canada

**DOI:** 10.36834/cmej.69332

**Published:** 2020-07-15

**Authors:** Kulamakan Kulasegaram, Melissa Hynes, Glen Bandiera, Patricia Houston

**Affiliations:** 1 The Wilson Centre & Department of Family & Community Medicine, University of Toronto, Ontario, Canada; 2 PostMD Education, University of Toronto, Ontario, Canada; 3 Department of Medicine, University of Toronto, Ontario, Canada; 4 MD Program, University of Toronto, Ontario, Canada; 5 Department of Anaesthesia, University of Toronto, Ontario, Canada

## Abstract

**Introduction:**

The MSPR is a Canada wide tool that provides aggregate information on MD students’ performance during training and used widely as part of PG admissions. This survey study elicits the perceptions of PG admissions stakeholders on the current use and future utility of the MSPR in Canada.

**Methods:**

PG admissions stakeholders across the faculties of medicine were convenience sampled for a 15-question online survey in the fall of 2018. Participants were asked how and when the MSPR is incorporated into the admissions process and perceptions and recommendations for improvement. Data are summarized descriptively and thematically.

**Results:**

Responses came from 164 participants across the 17 faculties of medicine. The MSPR was widely used (92%), most commonly in the file review process (52%) for professionalism issues. The majority of responses indicated that MSPRs were not fair for all MD students (60%) and required revision (74%) with greater emphasis required on transparency, professionalism, and narrative comments.

**Discussion:**

The results indicate that though MSPRs are widely used in PG admissions their perceived value is limited to a few specific sources of information and to specific parts of the admissions process. There are significant concerns from PG stakeholders on the utility of MSPRs and future changes should align with the needs of these stakeholders while balancing the concerns of students and undergraduate programs.

## Abstract (fr)

**Introduction:**

Le DREM est un outil pancanadien qui procure des renseignements regroupés sur le rendement des étudiants en médecine lors de la formation et il est largement utilisé dans le cadre des admissions post-doctorales. Cette étude par sondage révèle les perceptions des parties prenantes dans les admissions aux études médicales post-doctorales sur l’utilisation actuelle et l’utilité future du DREM au Canada.

**Méthodes:**

Les parties prenantes impliquées dans les admissions aux études médicales post-doctorales de l’ensemble des facultés de médecine ont été échantillonnés par convenance pour un sondage en ligne de 15 questions à l’automne 2018. Nous avons demandé aux participants comment et quand le DREM est intégré dans le processus d’admission et leurs perceptions et recommandations pour des améliorations. Les données sont résumées de manière descriptive par thèmes.

**Résultats:**

Les réponses proviennent de 164 participants provenant des 17 facultés de médecine. Le DREM a été largement utilisé (92 %) plus communément dans le processus d’examen du dossier (52 %) pour des questions de professionnalisme. La plupart des réponses indiquaient que les DREM n’étaient pas justes pour tous les étudiants en médecine (60 %) et nécessitaient une révision (74 %) avec une attention particulière sur la transparence, le professionnalisme et les commentaires narratifs.

**Discussion:**

Les résultats indiquent que bien que les DREM soient largement utilisés dans l’admission aux études médicales post-doctorales, leur valeur perçue est limitée à quelques sources particulières de renseignements et à des parties précises du processus d’admission. Il existe des préoccupations importantes des parties prenantes aux études post-doctorales sur l’utilité des DREM et les changements futurs devraient correspondre aux besoins de ces parties prenantes, tout en équilibrant les préoccupations des étudiants et des programmes de premier cycle.

## Introduction

The assessment of applicants for post-graduate (PG) admissions incorporates several sources of data,^1^ including the Medical Student Performance Record (MSPR). The MSPR summarizes aggregate academic achievement of medical students in Canada – similar to the Dean’s Letter in the United States.^2^ Its primary role is to testify to the academic performance of students and is thus a piece of information available for PG admissions committees to use for selection purposes. In content, format, and length, the MSPR varies greatly from school to school with some institutions providing summative marks, others providing narrative comments and the remainder a mix of both. The MSPR is sent to PG programs to which medical (MD) students have applied, and the programs are free to decide how best to incorporate this information into their decisions for selection.^1^ This freedom is appropriate given the wide variety of MSPR formats. However, this freedom and variation can create unique challenges when used for selection.

The role of the MSPR based on its questionable utility and value is being reconsidered as are other elements of the selection process. In 2018, a national committee was struck to evaluate the role of the MSPR and to explore harmonization of the tool.^2^ Any changes to the MSPR must be grounded in evidence of its utility and the value it provides to the immediate stakeholders: MD students,^3^ MD schools, and PG programs. Previous research has documented the variable content and structure of MSPRs across MD schools though little has been done to examine 1) how MSPRs are used in PG admissions, 2) the perceptions of utility, and 3) suggestions for the future optimization of the tool.^4^^-^^6^ In particular, the perceptions of utility from PG admissions committees are conspicuously absent or out of date in the Canadian literature.^5^ Any comprehensive and national changes to advance the MSPR and optimize its utility must be aligned with how PG stakeholders including Program Directors (PD), admissions committees, and other individuals currently use this tool and may wish to use it in the future.

To fill this gap, we conducted a national survey of PG leaders directly responsible for admissions. We asked how the MSPR was being used in the admissions process, as well as its perceived utility including strengths, weaknesses, and suggestions for improvement. Below we report on the findings and discuss the implications for ongoing changes to the MSPR lastly future directions for evaluation and scholarship.

## Methods

This national survey study received REB approval at the University of Toronto (#00036467). The target population included past and present individuals responsible for PG admissions. Given the varied structures of PG programs, the role for each individual was not specified. In the spring and summer of 2018, we created a survey to understand how the MSPR was being used and the perceptions of its utility.

KK and PH drafted the initial survey based on the work of the MSPR working group, specifically focusing on the gaps this group had identified.^2^ GB and MH reviewed and modified the survey. Based on feedback from two program directors at the University of Toronto, the refined survey included seven questions on how the MSPR is used, five Likert type questions on perceptions of the MSPR’s utility, 2 questions requesting open-ended feedback on the strengths and weaknesses of the MSPR, and 6 demographic questions (2 of 6 questions being used to exclude participants who were not currently or previously involved in PG admissions, and to exclude programs that do not use the MSPR as they would not be able to speak to MSPR use) (See Table 1 for final questions).

**Table 1:** Questions

Question	Type of Question
Do you currently use the MSPR as part of the CaRMs selection process for your program?	Yes/No (for inclusion purposes)
*Use questions*	Multiple response
At what stage do you use the MSPR?	
How do you use the MSPR?	
Who evaluates the MSPR?	
How is the MSPR evaluated?	
What do you believe the MSPR provides?	
What information do you value in the MSPR?	
In addition to the standard components of the CaRMs files, what other sources of data you use?	
*Utility questions*	
The MSPR is a useful tool during the CaRMs match	7-point Likert scale expressing Agreement
MSPRs from across different Canadian medical schools are equally useful	
The MSPR is a fair tool for candidates across all Canadian medical schools	
The MSPR should be standardized across all Canadian medical schools.	
The MSPR needs to be revised	
*List & Narrative Questions*	
Please list 3 features of the MSPR you like	Free form text
Please list 3 suggestions to improve the MSPR	

We used a professional translation service to translate the survey from English to French and then further reviewed it for coherence and clarity.

We used a combination of convenience and snowball sampling strategies with the English and French surveys being disseminated to the PG deans at all 17 medical schools in Canada using the Association of Faculties of Medicine postgraduate dean’s list serv. We asked the PG deans to forward the survey to all their PDs along with an accompanying explanatory email indicating the purpose, the intended recipients, and instructions for completion. The survey was sent using anonymous survey links on the Qualtrics platform; distribution took place in September 2018 and included 2 reminders. The survey was closed in November 2018 and data were extracted for analysis.

**Figure 1:**
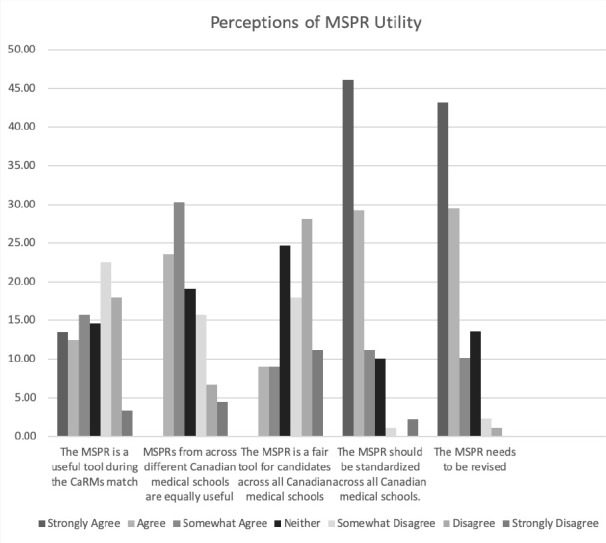
Perceptions of MSPR utility

We excluded data from respondents that indicated they were not currently or previous part of PG admissions. We summarized data using descriptive statistics and expressed results as percentages. For ease of interpretation we discuss the results by aggregated Strongly Agree, Agree, and Somewhat Agree into one category though more detailed results are presented in Figure 1. Additionally, we transformed the categories into numerical scores (1=Strongly Agree, 2=Agree etc.) to determine the modal response as a measure of central tendency. We coded open-ended comments for the most frequently expressed ideas or suggestions using an inductive and descriptive coding framework. As survey comments offer only brief narrative data, we focus only on the description of the themes and report any themes that had more than 10 individual instances. Inferential statistics were not considered appropriate as we had no a priori hypotheses about associations and the goal of the survey was descriptive, not inferential.

## Results

We received 182 responses to the survey, of which 118 responses to the English survey and 48 responses to the French survey were complete; two respondents were excluded as they indicated their program did not use the MSPR for a total of analyzable sample of 164 responses. Responses included representation from across the 17 Canadian medical schools though the number of responses per school ranged from two to 23. A large plurality came from Family Medicine (25%) with 11.9% from Medicine, 8% from Pediatrics, 8.4% from Surgery, 7% from Pathology/Lab medicine, and 6% from Psychiatry. All other specialities were under 5% with the exception of the ‘Other’ category which accounted for 14.3%. of our data. The majority of respondents (63%) had greater than 6 years of experience in PG admissions and 48% were PDs with other respondents having various roles in PG admissions. Nearly half of all respondents came from programs with 10 residents or less (48.8%) and about 37% came from programs with between 11 and 75 residents.

### How and when the MSPR is used

The majority of respondents recorded using the MSPR in CaRMS selection (92%). The primary use for the MSPR was during file review (52%), with approximately 8% reporting use prior to file review, during candidate interviews, or after candidate interviews. Just over 10% of respondents used the MSPR to screen out applicants prior to interviews and 14.5% reported using the MSPR to create rank lists for interviews. A small percentage reported using the MSPR through all stages of the application process including Round 2 of the match process (i.e. applications that were unmatched in first iteration). A large number of respondents indicated they use the MSPR primarily for professionalism screening (34%) and/or academic performance data. The most commonly valued components of the MSPR were the Narrative Comments and Professionalism information by a large margin (42% and 35% respectively) with years in program and other information found to be less useful. A few respondents commented that they looked for ‘red flags’ on academic and professional issues in the MSPRs. Respondents reported that MSPRs were reviewed by Faculty (56%), PDs (34%) and, less commonly by Fellows or Residents (16%).

### Perceptions of MSPR utility

Among respondents, approximately 51.1% disagreed to some extent that the MSPR was a useful tool for selection while 49.9% expressed agreement to some extent that it was (see Figure 1 for details). The modal response was ‘Neither Agree or Disagree’ indicating a lack of consensus on this issue with mode. Nearly 69% of respondents expressed some level of agreement that MSPRs were not equivalent across Canadian medical schools (mode: ‘Somewhat Agree’) and a similar proportion expressed interest in standardization (71%) (mode: ‘Strongly Agree’). Moreover, nearly 60% of respondents expressed some level of disagreement that the MSPR was a fair tool for all candidates (mode: ‘Disagree’). And the majority of all respondents (74%) Somewhat Agreed, Agreed, or Strongly Agreed that the MSPR needed revision. (mode: ‘Strongly Agree’)

From the open-ended free form data, the most commonly valued components of the MSPR were Narrative Comments and Professionalism information. Common suggestions for improvement included **Need for Standardization**, greater **Transparency** of how information was generated, more information on **Professionalism** issues, and more **Narrative comments** to convey this information. To a lesser extent, respondents called for more descriptive reporting including **Negative** information. A common modifier to recommendations for more narrative comments was the need for synthesis of information so that assessors can easily understand and interpret narrative comments in the MSPR.

## Summary

This survey was intended to describe how the MSPR was used in PG selection and elicit perceptions for utility. Our survey is limited as it was a convenience sample and we could not adequately estimate the appropriate denominator for the sampling population. Given the complexity of administrative structures for selection committees and processes, we aimed to gather input from multiple disciplines and institutions across Canada. While our survey only reports perception, it provides an important voice to a diverse and national group of stakeholders and end users of the MSPR.

Our results show the MSPR is utilized predominantly in the pre-interview process of the selection process and that the MSPR is used primarily as a screen for professionalism and academic information. Professionalism is a concern during PG training that requires admissions screening. MD programs may need to help PG admissions committees understand how professionalism is evaluated and reported during training. Respondents agreed with concerns about equivalency across Canadian medical schools and the need for greater standardization. Respondents overwhelming expressed a desire for change to the MSPR to include more information about professionalism, performance, and useful narrative data. At the same time, they were cognizant of the need for presenting information in synthesizable and interpretable ways. These suggest some overall utility in the MSPR while pointing to important steps in revising and improving the utility of the MSPR for future use in the CaRMS process. Still, representing complex constructs such as performance and professionalism via a single tool will be a challenging assessment problem

Our results empirically demonstrate both concerns about and proposals for improving the MSPR. Moving forward, continuing to engage PDs in the design and improvement of the MSPR is necessary as these individuals can provide information on the validity and utility of this tool. At the same time, balancing the desires of other stakeholders such as MD students and MD schools for ease of generation, fairness, privacy, and other concerns must not be forgotten. It is now more than ever imperative for rich competency-oriented data to assist in the selection of MD students for and their transition to PG training.
